# Supplementary material for: Identification of photoperiod-induced specific miRNAs in the adrenal glands of Sunite sheep (Ovis aries)
Source: Front Vet Sci. 2022 Jul 22;9:888207. doi: 10.3389/fvets.2022.888207 (PMC9354845; doi:10.3389/fvets.2022.888207)
Supplement: Supplementary file 1 [file Data_Sheet_1.ZIP › Supplementary file/miRDeep2 file.zip!/miRDeep2 file/Know_Structure/LP42A2/oar-mir-379.pdf]

miRBase precursor : oar-mir-379
Total read count : 44868
oar-miR-379-5p read count : 43494
oar-miR-379-3p read count : 1332
remaining reads : 42

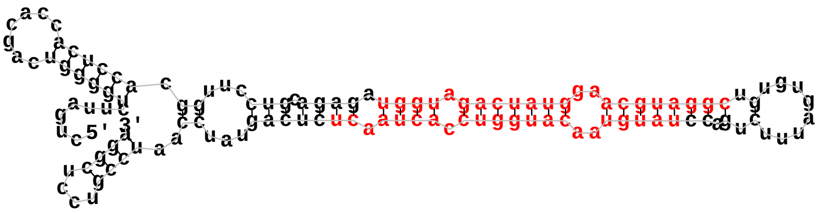

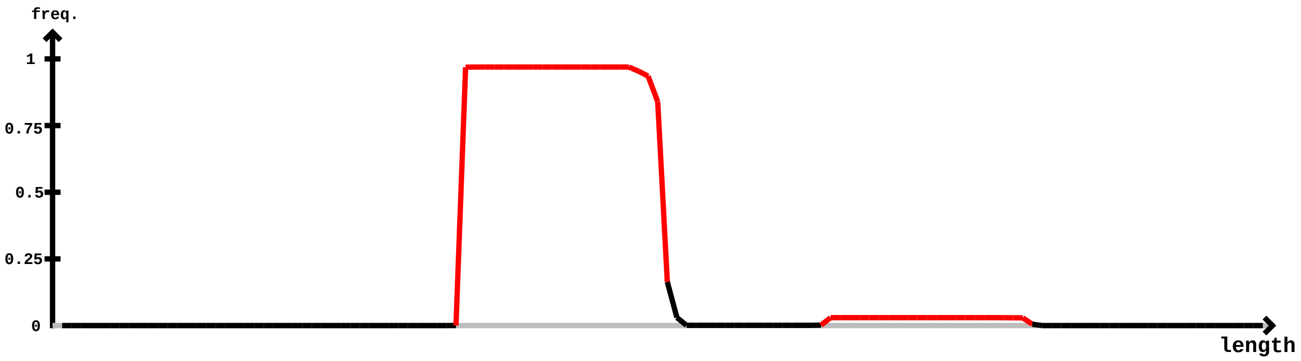


```
                                                                                    oar-miR-379-3p
                                         oar-miR-379-5p
5'- cugauucuggggucagcaccacuccacgguuccugcagagaugguagacuauggaacguaggcugugugauuucugaccuauguaacaugguccacuaacucucaguauccaauccguccucgga  -3'   exp
    .......((((((.......)))))).((...(((.((((.((((.(((((((..((((((((.(........).).)))))))..))))))).)))).)))))))...))..((((....))))   reads   mm
    .....................cuccacgguuccugcagaga....................................................................................   1       0
    ......................uccacgguuccugcagaga....................................................................................   1       0
    ........................................augguagacuauggaacguag................................................................   2       0
    ........................................augguagacuauggaacguagg...............................................................   9       0
    .........................................ugguagacuauggaacg...................................................................   5       0
    .........................................ugguagacuauggaacgu..................................................................   688     0
    .........................................ugguagacuauggaacgua.................................................................   836     0
    .........................................ugguagacuauggaacguag................................................................   4286    0
    .........................................ugguagacuauggaacguagg...............................................................   30345   0
    .........................................ugguagacuauggaacguaggc..............................................................   6000    0
    .........................................ugguagacuauggaacguaggcu.............................................................   1296    0
    .........................................ugguagacuauggaacguaggcug............................................................   2       0
    ..........................................gguagacuauggaacgu..................................................................   1       0
    ..........................................gguagacuauggaacgua.................................................................   1       0
    ..........................................gguagacuauggaacguag................................................................   3       0
    ..........................................gguagacuauggaacguagg...............................................................   14      0
    ..........................................gguagacuauggaacguaggc..............................................................   4       0
    ..........................................gguagacuauggaacguaggcu.............................................................   2       0
    ..............................................................cugugCgauuucugacc..............................................   1       1
    ..............................................................cugugugauuucugacc..............................................   18      0
    ..............................................................cugugugauuucugacU..............................................   20      1
    ..............................................................cuUugugauuucugacc..............................................   1       1
    ..............................................................................cuauguaacauggucc acu............................   1       0
    ..............................................................................cuauguaacaugguccacua...........................   1       0
    ..............................................................................cuauguaacaugguccacuaa..........................   2       0
    ..............................................................................cuauguaacaugguccacuaac.........................   20      0
    ..............................................................................cuauguaacaugguccacuaacu........................   3       0
    ...............................................................................uauguaacaugguccacC............................   1       1
    ...............................................................................uauguaacaugguccacA............................   1       1
    ...............................................................................uauguaacaugguccacu............................   6       0
    ...............................................................................uauguaacaugguccacua...........................   11      0
    ...............................................................................uauguaacaugguccacuaa..........................   14      0
    ...............................................................................uauguaacaugguccacuaac.........................   1042    0
    ...............................................................................uauguaacaugguccacuaacu........................   228     0
```

oar-miR-379-3p

oar-miR-379-5p

cugauucuggggucagcaccacuccacgguuccugcagagaugguagacuauggaacguaggcugugugauuucugaccuauguaacaugguccacuaacucucaguauccaauccguccucgga

....auguaacaugguccacuaac.........................	1	0
....auguaacaugguccacuaacu........................	1	0
